# Evaluation of an active live yeast (Levucell *Saccharomyces cerevisiae*, CNCM l-1077) on receiving and backgrounding period growth performance and efficiency of dietary net energy utilization in low health risk beef steers^1^

**DOI:** 10.1093/tas/txaa127

**Published:** 2020-07-17

**Authors:** Zachary K Smith, Kip Karges, Angel Aguilar

**Affiliations:** 1 Department of Animal Science, South Dakota State University, Brookings, SD; 2 Lallemand Animal Nutrition, Milwaukee, WI

**Keywords:** beef, receiving, *Saccharomyces cerevisiae*, steers

## Abstract

The objective of this experiment was to evaluate the influence of an active live yeast direct-fed microbial (DFM) product on receiving and backgrounding period growth performance and efficiency of dietary net energy (NE) utilization in low health risk beef steers. Maine-Anjou × Angus steers (*n* = 199; body weight [BW] = 252 ± 32.1 kg) were received from two sources at the Ruminant Nutrition Center in Brookings, SD, in November 2019 and used in a 77-d feedlot receiving and backgrounding experiment. Steers were provided access to long-stem hay and ad libitum water upon arrival. Steers were weighed, vaccinated for respiratory pathogens (source 2 only): infectious bovine rhinotracheitis, bovine viral diarrhea types 1 and 2, parainfluenza-3 virus, and bovine respiratory syncytial virus (Bovi-Shield Gold 5, Zoetis, Parsippany, NJ) vaccinated for clostridial species (Ultrabac 7/Somubac, Zoetis) and pour-on moxidectin (Cydectin, Bayer, Shawnee Mission, KS). Steers (*n* = 176 steers; initial unshrunk BW = 235 ± 27.6 kg) were allotted to pens (*n* = 20 pens; 10 pens per treatment; eight or nine steers per pen). Diets were based upon corn silage, dry-rolled corn, and dried distillers grains; dietary treatments were 1) no DFM (CON) and 2) DFM (Levucell SC, Advantage Titan, CNCM l-1077), fed at 10 g/steer/d providing 8 × 10^9^ CFU of active live yeast to each steer daily (DFM). Initial BW was the average of day −1 and day 1 BW (*n* = 176 steers; initial BW = 253 ± 27.6 kg). On day 21, steers received a 200-mg progesterone and 20-mg estradiol benzoate implant. Data were analyzed from day 1 to 47 (receiving period), day 48 to 77, and from day 1 to 77 as a randomized complete block design; pen served as the experimental unit for all analyses. On day 47 of the experiment, DFM had greater BW (*P* = 0.01) by 0.9% and average daily gain (ADG; *P* = 0.01) by 4.2% and gain-to-feed ratio (G:F) tended (*P* = 0.13) to be 2.8% greater. Day 77 BW did not differ (*P* = 0.60), cumulative (days 1–77): ADG (*P* = 0.47), dry matter intake (*P* = 0.66), and G:F (*P* = 0.56) were similar. Yeast inclusion had no appreciable influence on performance-based dietary NE utilization or the ratio of observed/expected dietary NE (*P* ≥ 0.59). In low health risk steers, DFM improved performance during the feedlot receiving period. However, no improvements for DFM were detected for cumulative performance from day 1 to 77. The confirmation of yeast counts indicated the CFU to be above the expected level at the start of the trial but was found below expected level at the end of the trial. This may explain differences during the initial 47 d compared to cumulative growth performance results.

## INTRODUCTION

In conventional beef production systems, feed-grade antimicrobials are used to reduce metabolic disorders and improve feed conversion efficiency (Smith et al., 2020). Feed-grade antibiotics have been used in beef cattle production for over 60 yr (Landers et al., 2012). The current Veterinary Feed Directive and implementation of all-natural beef marketing channels have created the need for nonantimicrobial feed additives that have the potential to improve feed conversion efficiency, aid in ruminal fermentation, and improve animal health outcomes. Feeding enzymatically hydrolyzed yeast, yeast culture products, and active live yeast strains of *Saccharomyces cerevisiae* (SC) to cattle has produced inconsistent results in relation to gain, intake, gain efficiency, and efficiency of dietary net energy (NE) utilization (Zinn et al., 1999; Finck et al., 2014; Salinas-Chavira et al., 2015; Kayser et al., 2016; Ovinge et al., 2018; Salinas-Chavira et al., 2018). The objective of this experiment was to evaluate the influence of an active live yeast product (Levucell SC, Advantage Titan, CNCM l-1077, Lallemand Animal Nutrition, Milwaukee, WI) on receiving and backgrounding period growth performance and efficiency of dietary NE utilization in low health risk beef steers. We hypothesized that feeding an active live yeast would increase gain, intake, and the efficiency of dietary NE utilization in low health risk beef steers.

## MATERIALS AND METHODS

Animal care and handling procedures used in this study were approved by the South Dakota State University Animal Care and Use Committee (approval number: 1910-058E).

### Dietary Treatments

This study used 10 replicate pens (20 pens total) of eight or nine steers per pen assigned to one of two dietary treatments in a randomized complete block (blocked by batch fraction) design (Blom et al., 2020; Gentry et al., 2020). The dietary treatments were incorporated into the total mixed ration using a soybean hull pelleted supplement that was manufactured in October of 2019 (2 weeks prior to the initiation of the experiment) at the South Dakota State University Feed Mill in Brookings, SD, and stored under cover at ambient temperature at the research facility in galvanized bins.

Treatments included no direct-fed microbial (DFM) contained in the diet (CON) and a diet that contained the experimental SC DFM (Levucell SC, Advantage Titan, CNCM l-1077 at 10 g/steer/d providing 8 × 10^9^ CFU, Lallemand Animal Nutrition; DFM).

### Enumeration of Yeast Count and Confirmation of SC

A quantitative enumeration of SC yeasts was performed under aerobic conditions. All treatment supplement samples were analyzed in duplicate. Ten grams of the yeast supplement were weighed and transferred into a blender bowl and a tempered dilution solution containing 8.5 g of NaCl, 2.5 g of K_2_HPO_4_, 2.5 g of KH_2_PO_4_, 1.0 g of casein peptone, and 1 g of polysorbate 80 that was taken to a volume of 1 L using demineralized water and maintained at 37 °C was added up to 100 g along with the yeast supplement. Next, 0.4 mL of polypropylene glycol was added as an antifoaming agent since the yeast product was microencapsulated. The resulting suspension was blended for 1 min and then transferred into a flask and allowed to rehydrate for 15 min at 37 °C with gentle agitation (120 rpm) in a shaker water bath. Next, the rehydrated suspension was transferred to a stomacher bag with a filter and homogenized for 1 min at maximum speed resulting in a 10 to 1 dilution. Then 1 mL of the resulting 10 to 1 dilution was added to 9 g of dilution medium that contained 1 g casein peptone and 8.5 g of NaCl that was brought to a volume of 1 L using demineralized water and repeated until the desired dilution was obtained. Then, approximately 200 μL of the dilution was plated in triplicate, spread using an auto plater, inverted, and then incubated at 30 °C for 48 h. Yeast colonies were counted and total cell count (CFU) per gram was determined according to the following equation: $\frac{Number\;of\;colonies\;in\;the\;3\;plates\;\times \;dilution\;factor}{3}$.

The confirmation of yeast counts in the pelleted treatment supplement indicated the CFU to be at 1.3 × 10^8^ cfu/g, which was above the expected level of 8.5 × 10^6^ cfu/g at the start of the trial but was found to be reduced to 1.1 × 10^6^ cfu/g at the end of the trial, which was 90 d after the pellet was initially manufactured.

### Animal and Feeding Management

One hundred and ninety-nine low health risk Maine-Anjou × Angus beef steers (252 ± 32.1 kg) from two sources were shipped 64 and 199 km, for source 1 and source 2 steers, respectively, and received at the Ruminant Nutrition Center (RNC) in Brookings, SD, in November of 2019. Upon arrival to the RNC, steers were housed (*n* = 8–10 steers/pen) in 7.62- × 7.62-m concrete surface pens with 7.62 m of linear bunk space and provided ad libitum access to long-stem grass hay and water. The following day, all steers were individually weighed (scale readability ± 0.454 kg), applied a unique identification ear tag, vaccinated for viral respiratory pathogens (source 2 steers only as all source 1 steers had been vaccinated prior to arrival): infectious bovine rhinotracheitis, bovine viral diarrhea types 1 and 2, parainfluenza-3 virus, and bovine respiratory syncytial virus (Bovi-Shield Gold 5, Zoetis, Parsippany, NJ), both sources were vaccinated for clostridial species (Ultrabac 7/Somubac, Zoetis) and administered pour-on moxidectin (Cydectin, Bayer, Shawnee Mission, KS) according to label instructions. The afternoon following initial processing, all steers were allotted to their study pens (*n* = 8–9 steers/pen and 10 pens/treatment). The following morning, all steers were again individually weighed and experiment was initiated. Steers used (*n* = 176) were selected for uniformity from the pool of 199 steers. The initial body weight (BW) was the average of processing BW (day −1 BW) and day 1 BW (*n* = 176 steers; initial unshrunk BW = 253 ± 27.6 kg); an equal number of steers from each source were enrolled to each treatment in the experiment. On day 21, all steers were implanted with 200 mg progesterone and 20 mg estradiol benzoate (Synovex-S, Zoetis).

Receiving and backgrounding diets (Table 1) were formulated to provide vitamins and minerals to meet or exceed nutrient requirements and provided [dry matter (DM) basis] monensin sodium at 27.6 mg/kg of DM (NASEM, 2016). There was no morbidity or mortality noted in the present study. Fresh feed was manufactured twice daily in a stationary mixer (2.35 m^3^; readability ± 0.454 kg; Roto-Mix, Dodge City, KS) and offered to steers in two equal deliveries. Orts were collected, weighed, and dried in a forced-air oven (Despatch, Minneapolis, MN) at 100 °C for 24 h in order to determine DM content if carryover feed went out of condition or was present on weigh days; if carryover feed was present on weigh days, the residual feed was removed prior to the collection of BW measurements. The dry matter intake (DMI) of each pen was adjusted to reflect the total DM delivered to each pen after subtracting the quantity of dry orts for each interim period. Actual diet formulation is based upon weekly ingredient DM analyses (drying in a forced-air oven at 60 °C until no further weight change) and corresponding feed batching records. Diets presented in Table 1 are actual DM diet composition from weekly ingredient DM analysis, actual assayed nutrient concentrations from weekly commodity ingredient sampling for crude protein (CP), neutral detergent fiber (NDF), acid detergent fiber (ADF), ash, and ether extract (EE): method no. 968.06 (AOAC 2016) for CP using the Rapid Max N Exceed, Elementar, Mt. Laurel, NJ; NDF and ADF (Goering and Soest 1970); method no. 942.05 (AOAC 2012) for ash; and EE using petroleum ether, method no. 2003.06 (AOAC 2007), and tabular energy values (Preston, 2016).

**Table 1.** Actual diet formulations and nutrient composition of diets fed^*a*,*b*,*c*,*d*^

	Dietary treatment					
	Days 1–7		Days 8–21		Days 22–77	
Item	CON	DFM	CON	DFM	CON	DFM
Corn silage, %	38.07	38.07	37.28	37.28	60.14	60.14
Dry-rolled corn, %	17.22	17.22	19.88	19.88	15.63	15.63
Dried distillers grains plus solubles, %	14.66	14.66	14.88	14.88	15.73	15.75
Pelleted supplement, %^*e*^	5.88	5.88	6.10	6.10	6.40	6.40
Pelleted treatment supplement, %^*f*^	4.13	4.13	2.29	2.28	2.10	2.08
Grass hay, %	20.04	20.04	19.58	19.57	—	—
Nutrient composition						
DM, %	54.32	54.33	52.65	52.66	43.42	43.42
CP, %	12.43	12.40	12.74	12.73	12.39	12.39
NDF, %	37.75	37.75	36.50	36.50	32.64	32.64
ADF, %	28.14	28.14	27.11	27.11	18.74	18.73
Ash, %	6.72	6.72	6.05	6.05	5.84	5.84
EE, %	3.90	3.90	4.00	4.00	3.88	3.88
NE_m_, Mcal/kg	1.78	1.78	1.79	1.79	1.84	1.84
NE_g_, Mcal/kg	1.12	1.12	1.13	1.13	1.21	1.21

^*a*^Treatments included: no DFM contained in the diet (CON) and a diet that contained the experimental *Saccharomyces cerevisiae* DFM (Levucell SC, CNCM l-1077, Advantage Titan at 10 g/steer/d providing 8 × 10^9^ CFU, Lallemand Animal Nutrition; DFM).

^*b*^All values except DM on DM basis.

^*c*^Mean values based upon weekly ingredient assays and daily feed batching records.

^*d*^Dietary NE values based upon tabular feed values and true ingredient inclusion levels.

^*e*^Contained (as-is basis) per 907 kg: 613 kg of soybean meal, 91 kg of soybean hulls, 43 kg of trace-mineralized salt, 157 kg of calcium carbonate, 1,972 g of Rumensin-90 (Elanco, Indianapolis, IN), 48 g of vitamin A (650,000 IU/g), 750 g of vitamin E (500 IU/g), 721 g of intellibond Zn (Micronutrients, Indianapolis, IN), and 195 g intellibond Cu (Micronutients). Pellet averaged 89% DM.

^*f*^Contained (as-is basis) per 907 kg: 907 kg of soybean hulls (CON) or 847.7 kg of soybean hulls and 59.3 kg of Levucell SC, CNCM l-1077, Advantage Titan (Lot number: LA080820180220B; Lallemand Animal Nutrition) with a label guarantee of 8 × 10^8^ CFU/g and an assayed value of 12.9 × 10^8^ CFU/g (Levucell). Both pellets were 89% DM. Pellet DM inclusion level was altered to ensure equal intake of the pelleted treatment supplement across differing levels of DMI.

### Growth Performance Calculations

Steers were individually weighed on days −1, 1, 21, 47, and 77 using a hydraulic squeeze chute mounted on load bearing weight cells (scale readability ± 0.454 kg). Weight gain was based upon initial unshrunk BW (average of day −1 and day 1 BW), and day 77 BW was pencil shrunk 4% to account for gastrointestinal tract fill. Daily energy gain (EG, Mcal/d) was calculated according to the medium frame steer calf equation: EG = 0.0557BW^0.75^ × ADG^1.097^ (NRC, 1984). Energy gain was the daily deposited energy and BW was the average BW from the 77-d receiving period using initial unshrunk BW, and day 77 BW shrunk 4% (NRC, 1984, 1996). Maintenance energy (EM, Mcal/d) was calculated as: EM = 0.077BW^0.75^ (Lofgreen and Garrett, 1968; NASEM, 2016). Using the estimates required for maintenance and gain, the performance-based dietary NE_m_ and NE_g_ values, as illustrated by Owens and Hicks (2019), of the diet were generated using the quadratic formula: $x=\frac{-b\pm \sqrt{b^{2}-4ac}}{2c}$, where *x* = diet NE_m_, Mcal/kg, *a* = −0.41 EM, *b* = 0.877 EM + 0.41 DMI + EG, *c* = −0.877 DMI, and NE_g_ was determined from 0.877 NE_m_ −0.41 (Zinn and Shen, 1998; Zinn et al., 2008).

### Statistical Analysis

All data were analyzed using a model appropriate for a randomized complete block design experiment blocked by batch fraction fed from the mixer according to Blom et al. (2020) and Gentry et al. (2020) using the GLIMMIX procedure of SAS 9.4 (SAS Inst. Inc., Cary, NC), considering dietary treatment and block as fixed effects; no random effects were included in the model. Pen served as the experimental unit for all analyses. Treatment differences were evaluated by the pairwise differences and lines (PDIFF LINES) statement of SAS 9.4 (SAS Inst. Inc.). An *a* of 0.05 determined significance and an *a* of 0.06–0.10 was considered a tendency.

## RESULTS AND DISCUSSION

### Steer Growth Performance

No cattle were treated for respiratory ailments or subjected to further observation during the 77-d receiving and backgrounding period, and steers in the present study were not subjected to severe transit or marketing stress prior to arrival at the RNC. Others have noted that the use of a yeast product can decrease morbidity and days showing clinical signs of disease in shipping-stressed steers (Zinn et al., 1999), and others have noted that the use of a yeast product has no influence on overall morbidity in newly weaned beef steers that were subject to transit stress (Deters et al., 2018). There was no difference (*P* = 0.10) in the initial unshrunk BW (Table 2). On day 47, DFM had greater BW (*P* = 0.01) and ADG (*P* = 0.01) but DMI and G:F did not differ (*P* ≥ 0.13). Others have noted no differences in ADG (Zinn et al., 1999; Ovinge et al., 2018), while some have noted improvements in G:F when a yeast culture product was used in transit-stressed beef calves during feedlot receiving period (Zinn et al., 1999). No differences were detected (*P* ≥ 0.20) for any interim growth performance measures from day 48 to 77. When a live yeast *Saccharomyces cerevisiae boulardii* CNCM I-1079 was fed to high-risk receiving heifers, improvements in growth performance during the initial 45 d on feed, as well as improved health outcomes, were noted (Theurer et al., 2019). When an enzymatically hydrolyzed yeast was fed to growing Holstein steers, a 3.4 % increase in ADG and a 3.4 % increase in DMI was noted and steers exhibited similar feed efficiency (Salinas-Chavira et al., 2018). However, when enzymatically hydrolyzed yeast was fed to crossbred beef steers, a 9% increase in ADG was observed during the finishing phase of production (Salinas-Chavira et al., 2015). Inconsistencies in the literature compared to our study could be due to reduced yeast counts that were found in the pelleted treatment supplement at the end of the present experiment. The present experiment indicates that confirmation testing of yeast during the course of the feeding period should be done if live yeast products are to be manufactured in a single event and fed over a long period of time. In practice, most cattle feeding entities would ideally not keep feed inventory for greater than 30 d, so this artifact of the present experiment might not be an issue in a production setting.

**Table 2.** Interim (unshrunk) steer growth performance^*a*^

Item	CON	DFM	SEM	*P*-value
Pens	10	10	—	—
No. steers	88	88	—	—
Initial BW, kg^*b*^	253	252	0.2	0.10
Initial to day 47				
BW day 47, kg^*c*^	329	332	0.6	0.01
ADG, kg	1.62	1.69	0.015	0.01
DMI, kg	7.14	7.22	0.051	0.33
G:F	0.227	0.234	0.0027	0.13
Days 48–77				
BW day 77, kg^*c*^	365	366	1.3	0.60
ADG, kg	1.21	1.15	0.034	0.23
DMI, kg	7.98	7.93	0.058	0.58
G:F	0.152	0.146	0.0033	0.20

^*a*^Treatments included: no DFM contained in the diet (CON) and a diet that contained the experimental *Saccharomyces cerevisiae* DFM (Levucell SC, CNCM l-1077, Advantage Titan at 10 g/steer/d providing 8 × 10^9^ CFU, Lallemand Animal Nutrition; DFM).

^*b*^Average of BW from November 5, 2019 and November 6, 2019 was used as the initial BW (no shrink was applied to this BW).

^*c*^No shrink was applied to day 47 or day 77 BW.

There were no differences detected (*P* ≥ 0.47) for cumulative growth performance from initial to day 77 (Table 3). Final off-test BW (day 77 BW pencil shrunk 4% to account for digestive tract fill) did not differ (*P* = 0.60) between treatments. There was no difference (*P* = 0.47) for cumulative ADG detected in the present study. Cumulative DMI did not differ (*P* = 0.66) throughout the 77-d receiving and backgrounding experiment. This is similar to what others have observed in yearling beef steers when fed an active live yeast (Kayser et al., 2016; Ovinge et al., 2018). Cumulative G:F did not differ during the 77-d receiving and backgrounding period (*P* = 0.64) between treatment groups. Lack of responses to yeast supplementation on G:F responses is similar to what others have noted in yearling beef steers (Kayser et al., 2016). Finck et al. (2014) noted improvement in heifer DMI and health-related outcomes when fed live yeast and yeast cell wall alone or in combination compared to a nonsupplemented group during a 56-d receiving period. While differences in cumulative animal growth performance were not detected in this experiment, it is important to note that, in the present experiment, cattle had not been subjected to severe transit or marketing stress. Carryover improvements have been demonstrated in growth performance and subsequently improved carcass quality grade due to 45 d of live yeast supplementation during the initial receiving phase of production in high-risk heifers fed in a commercial feedlot (Theurer et al., 2019). Differential responses to yeast product supplementation on animal growth performance parameters might be explained by the degree of stress imposed upon incoming feeder cattle.

**Table 3.** Cumulative steer growth performance and efficiency of dietary NE utilization^*a*^

Item	CON	DFM	SEM	*P*-value
Pens	10	10	—	—
No. steers	88	88	—	—
Days on feed	77	77	—	—
Initial BW, kg^*b*^	253	252	0.2	0.10
Final BW, kg^*c*^	351	352	1.2	0.60
ADG, kg	1.27	1.29	0.016	0.47
DMI, kg	7.47	7.50	0.016	0.66
G:F	0.171	0.172	0.0020	0.64
Observed dietary NE, Mcal/kg				
Maintenance	1.83	1.84	0.014	0.70
Gain	1.19	1.20	0.013	0.59
Observed/expected dietary NE^*d*^				
Maintenance	1.00	1.01	0.008	0.70
Gain	1.00	1.01	0.011	0.59

^*a*^Treatments included: no DFM contained in the diet (CON) and a diet that contained the experimental *Saccharomyces cerevisiae* DFM (Levucell SC, CNCM l-1077, Advantage Titan at 10 g/steer/d providing 8 × 10^9^ CFU, Lallemand Animal Nutrition; DFM).

^*b*^Average of BW from November 5, 2019 and November 6, 2019 was used as the initial BW (no shrink was applied to this BW).

^*c*^A 4% pencil shrink was applied to day 77 BW to account for digestive tract fill.

^*d*^Actual trial NE based upon weighted average of diets fed were: 1.83 Mcal/kg of NEm and 1.19 Mcal/kg of NEg.

### Efficiency of Dietary NE Utilization

The influence of active live yeast supplementation on efficiency of dietary NE utilization is presented in Table 3. There were no appreciable differences detected for performance-based dietary NE_m_ (*P* = 0.70) or NE_g_ (*P* = 0.59) in the present study. This is similar to what others have reported in response to the use of a yeast culture-based product in shipping-stressed calves and growing–finishing Holstein steers (Zinn et al., 1999; Salinas-Chavira et al., 2018). Likewise, there were no differences detected between treatments for the ratio of observed to expected dietary NE_m_ (*P* = 0.70) or the ratio of observed to expected dietary NE_g_ (*P* = 0.59), which is similar to what others have demonstrated in a transit-stressed calf model, as well as growing and finishing Holstein steers (Zinn et al., 1999; Salinas-Chavira et al., 2018).

## CONCLUSIONS

In summary, DFM fortification of diets improved growth performance of low health risk steers (not transit or marketing stressed) during the feedlot receiving phase (initial 47 d). Positive effects of DFM inclusion into the diet were not detected for growth performance from day 48 to 77 or for cumulative growth performance from day 1 to 77. The confirmation of yeast counts showed that colonizing forming units were above the expected count at the start of the trial but were below the expected count at the end of the trial, which may explain the differences in growth performance observed during the initial 47 d compared to 48–77 d or cumulative growth performance.
